# Adjunct prednisone therapy for patients with community-acquired pneumonia: a randomized, placebo-controlled multicenter trial

**DOI:** 10.1186/cc14205

**Published:** 2015-03-16

**Authors:** CA Blum, N Nigro, M Briel, P Schuetz, E Ullmer, I Suter-Widmer, B Winzeler, R Bingisser, H Elsaesser, D Drozdov, B Arici, SA Urwyler, J Refardt, P Tarr, S Wirz, R Thomann, C Baumgartner, H Duplain, D Burki, W Zimmerli, N Rodondi, B Mueller, M Christ-Crain

**Affiliations:** 1 University Hospital Basel, Switzerland; 2 Medical University Clinic, Kantonsspital Aarau, Switzerland; 3 Kantonsspital Baselland/Liestal, Liestal, Switzerland; 4 Kantonsspital Baselland/Bruderholz, Bruderholz, Switzerland; 5 Bürgerspital, Solothurn, Switzerland; 6 Inselspital, Bern University Hospital, Bern, Switzerland; 7 Hôpital du Jura, Site de Delémont, Delémont, Switzerland; 8 Viollier SA, Basel, Switzerland

## Introduction

Clinical trials yielded conflicting data about the benefit of adding systemic corticosteroids for community-acquired pneumonia (CAP). We evaluated whether short-term corticosteroid treatment reduces time to clinical stability in patients hospitalized for CAP.

## Methods

This randomized, placebo-controlled multicenter trial compared prednisone 50 mg for 7 days with placebo in patients hospitalized with CAP. The primary endpoint was time to clinical stability.

## Results

Overall, 802 patients were randomized in seven Swiss hospitals from December 2009 to May 2014. Time to clinical stability was shorter in the prednisone group compared with placebo (3.0 vs. 4.4 days, HR = 1.33, 95% CI = 1.15 to 1.50, *P *< 0.001). The prednisone group as compared with the placebo group had a shorter time to hospital discharge (6 vs. 7 days (HR = 1.19, 1.04 to 1.38), *P *= 0.012) and a shorter duration of intravenous antibiotic treatment (4 vs. 5 days (difference, -0.89 days, -0.20 to -1.57 days), *P *= 0.011). All-cause mortality, ICU stay, recurrent pneumonia and rehospitalization rate were similar in both groups. Incidence of pneumonia-associated complications until day 30 tended to be lower in the prednisone group (2.8% vs. 5.6%, OR = 0.49, 0.23 to 1.02, *P *= 0.06). The prednisone group had a higher rate of in-hospital hyperglycemia needing insulin treatment (19.4% vs. 10.9%, OR = 1.96, 1.31 to 2.93, *P *= 0.001). Other adverse events compatible with corticosteroid use were rare and similar in both groups.

## Conclusion

Prednisone treatment for 7 days in hospitalized patients with CAP shortens time to clinical stability, time to hospital discharge and duration of intravenous antibiotic treatment without an increase in complications.

